# Parental Knowledge, Attitudes, and Behaviors Toward Their Epileptic Children at King Abdulaziz University Hospital: Cross-Sectional Study

**DOI:** 10.2196/12697

**Published:** 2020-01-20

**Authors:** Abdulelah Kinkar, Dalya Alqarni, Abdulaziz Alghamdi, Sahal Wali, Nasser Alghamdi, Saeed Saloom, Mooataz Aashi

**Affiliations:** 1 Faculty of Medicine, King Abdulaziz University Jeddah Saudi Arabia; 2 Department of Pediatrics King Abdulaziz University Hospital Jeddah Saudi Arabia

**Keywords:** parenting, attitudes, behaviors, epilepsy, children, pediatrics

## Abstract

**Background:**

Epilepsy is a chronic disease characterized by periodic seizures that result from abnormal integrated firing impulses in the brain. It is one of the most common neurological disorders. Over the past few years, there has been increasing awareness about the effect that having a child with epilepsy has on parents and the reciprocal impact of parental knowledge and attitudes regarding epilepsy on the affected child.

**Objective:**

This study aimed to assess parental knowledge, attitudes, and behavior toward their epileptic children.

**Methods:**

A cross-sectional study was conducted in 2018 by the Pediatric Neurology Department of King Abdulaziz University Hospital, Jeddah, the Kingdom of Saudi Arabia. A sample size of 115 of 332 parents who have a child diagnosed with epilepsy and aged 18 years or younger were recruited for this study. Statistical analysis was performed using SPSS version 21. Data analysis was performed using an independent *t* test, a chi-square test, one-way analysis of variance, and correlation analysis.

**Results:**

A total of 115 participants answered the questionnaire; of these, 65 (56.5%) were men, with an average age of 40.3 years, and the mean age of the children was 9.0 years. Overall, 85 (85/115, 73.9%) children were taken care of by both of their parents. The mean parental knowledge score was 7.49 (SD 2.08) out of 12, and it was significantly related to the educational level of the parent (*P*=.004). The knowledge question that was most frequently answered incorrectly was “Diagnosis of epilepsy is usually made based on at least two unprovoked seizures.” As only 28.7% (33/115) of participants chose the correct answer, mean parental attitude score was 26.51 (SD 4.284) out of 35, and there was no significant relation with the educational level of parents (*P*=.13); however, it was negatively correlated with the child’s age (*P*=.045). Mean parental behavioral score was 23.35 (SD 4.121) out of 35, and there was no significant relation with the educational level of the parents (*P*=.24). The most negatively answered question for the behavior section was “I can leave my child without supervision,” with a mean score of 2.25 (SD 1.09) out of 5. Gender did not play a significant role in parental knowledge, attitudes, or behavior (*P*=.44, *P*=.77, and *P*=.99, respectively).

**Conclusions:**

Parental knowledge in our sample still needs improvement. Therefore, more awareness campaigns should be made for the community and for the parents of affected children to create a supportive environment for the children and help them thrive and develop.

## Introduction

Epilepsy is a chronic disease characterized by periodic seizures that result from abnormal integrated firing impulses in the brain [1]. It is one of the most common neurological disorders [2]. The primary cause of epilepsy is unknown, but brain tumors and other diseases may cause epilepsy [3]. Most seizures in children are predisposed by disorders from outside the brain, such as high fever, infection, and trauma, and sometimes by genetic diseases [4,5].

Around 50 million people have epilepsy worldwide, which accounts for 1% of the global burden of disease [6]. Among Arab countries, it is demonstrated that around 724,500 persons had epilepsy. A survey was conducted in Saudi Arabia to determine the prevalence of epilepsy and other convulsive disorders, and it found that the rate of active epilepsy was 6.54 per 1000 persons (95% CI 5.48-7.60) [7], where 65% were patients whose epilepsy started before the age of 18 years [1]. Thus, in children, the dilemma of epilepsy is more severe than in adults; therefore, up to 50% of children with epilepsy might have psychiatric and behavioral comorbidities, such as learning disabilities, developmental delay, and autism spectrum disorders [8].

Public attitudes and knowledge toward epilepsy differ from one culture to another; for example, convulsive episodes at an unexpected moment in public may result in discrimination toward someone who is suffering from epilepsy [9-16]. It has been noted that traditional thoughts and poor knowledge strongly affect attitudes toward epilepsy [5,9]. Over the past few years, there has been increasing awareness about the effect of having a child with epilepsy has on parents and the reciprocal impact of parental knowledge and attitudes regarding epilepsy on the affected child [17,18]. Besides, parental attitudes toward epilepsy were as significantly associated with the child outcome as seizure history and epilepsy duration [19]. In addition, it is related to their psychosocial issue [15] because taking care of a child suffering from epilepsy results in a more significant amount of stress, which is associated with exaggerated fears and protective behaviors toward their children, that results in poor child behavioral outcome, compared with taking care of healthy child [19].

On reviewing multiple publications in Saudi Arabia, we concluded that there is little information related to this study; therefore, we aimed to study and evaluate parental knowledge, attitudes, and behavior toward their epileptic child and conclude whether further awareness programs are essential to improve the overall quality of life for epileptic children and their caregivers.

## Methods

### Study Design

The institutional review board of King Abdulaziz University (KAU) Hospital approved this study. A cross-sectional study assessing parental knowledge, attitudes, and behavior toward their epileptic children was conducted in 2018 by the Pediatric Neurology Department of KAU Hospital, Jeddah, the Kingdom of Saudi Arabia. A sample size of 115 of 332 parents who have a child diagnosed with epilepsy and aged 18 years or younger were recruited for this study. The data collected through telephone interview or Web-based Google form questionnaire, which was modified from international surveys that were previously used to assess parental knowledge and attitudes toward epilepsy, were translated into Arabic and then back translated to make sure the translation was accurate. Those who agreed to participate provided verbal consent.

Microsoft Excel version 2013 was used for data entry, and statistical analysis was performed using IBM SPSS Inc version 21. Data analyses were performed using an independent *t* test, a chi-square test, one-way analysis of variance, and correlation analysis.

The questionnaire contained two segments: sociodemographic characteristics of the parents (eg, gender, age, nationality, and educational degree) and demographic characteristics of their children with epilepsy (eg, age, educational level, child performance in school, the age of diagnosis, and who is the caregiver of the child).

### Parental Knowledge Toward Epileptic Children

The knowledge section of the questionnaire contained 12 items, 10 of which were yes or no or I don’t know questions. These questions included whether epilepsy is contagious, psychological, or genetic; whether or not all epileptic children lose consciousness during seizures; do all of the affected children show the same symptoms; is it possible for epileptic children to take vaccine according to a specific timetable; whether or not epileptic children had lower IQ than their peers; and whether or not epileptic children had difficulties in learning. The last 2 yes/no/I don’t know questions were regarding the use of medications—whether or not the child is cured of seizures after using medicine and whether or not, after forgetting to take the prescribed drug, the patient must double the dose next time. The other 2 questions had multiple choices: age of onset of seizures (before 1 year of age/until 18 years of age/at any age) and what indicates the diagnosis of epilepsy (loss of consciousness during a seizure/one unprovoked seizure/at least two unprovoked seizures).

Each correct answer was given 1 point. Therefore, the total score of knowledge was graded between 0 (the lowest grade) and 12 (the highest grade) points.

### Parental Attitudes Toward Epileptic Children

A total of 7 statements were scored using Likert scales (summative scales; strongly disagree, disagree, neither agree nor disagree, agree, and strongly agree), where the minimum score 1 represented “I strongly disagree” and the maximum score 5 represented “I strongly agree.” The total score ranged from 7 to 35. Parents were asked about whether they feel comfortable if their family or friends knew that they have an epileptic child; if they would be worried about their child capability on making friends; if, perhaps, other people will treat their child differently; if they believe that their child will be able to achieve success in his/her career; if they believe their child will be as qualified as any other healthy children or, if there is more social support, the child will have more chances to self-develop; and if they receive enough support from the society for their epileptic child.

### Parental Behavior Toward Epileptic Children

A total of 7 statements were scored using Likert scales (strongly disagree, disagree, neither agree nor disagree, agree, and strongly agree), where the minimum score 1 represented “I strongly disagree” and the maximum score 5 represented “I strongly agree.” The total score ranged from 7 to 35. Parents were asked about whether they are capable of taking care of their child during seizures, if they allow their child to participate in any activity he/she desires, if they would let their child to play video games or play a sport or to go on school trips, if they can leave their child without supervision, and if the parents have time to meet other people and participate in other activities for themselves.

## Results

This study aimed to evaluate parental knowledge, attitudes, and behaviors toward their epileptic children.

Of the 115 study participants, 65 (56.5%) were men. The average parental age was 40.3 years (SD 8.4; age range: 24-63 years). Overall, 77 (67.0%) were Saudi parents. In addition, 54 (47.0%) parents received a college education, and 4 (3.5%) did not receive any education.

Almost three-fourth of the children were taken care of by both of their parents (85/115, 74.0%). The mean age of the children was 9.0 years (SD 5.3; age range: 1-18 years). Moreover, 51 (44.3%) children were diagnosed with epilepsy before the first year of life.

Other sociodemographic characteristics of parents and children are presented in Table 1.

The mean parental knowledge score was 7.49 (SD 2.08). Of 115 parents, 104 (90.4%) knew that epilepsy is not a contagious disease. In contrast, only 33 (28.6%) knew how epilepsy is diagnosed. There was no significant difference in the knowledge scores between men (mean score 7.35) and women (mean score 7.66; *P*=.44). Education had a statistically significant effect; parents with more than 12 years of schooling scored higher in knowledge (mean score 8.09) than those who spent less than 12 years (mean score 6.97; *P*=.004). There was no significant difference in the knowledge scores between Saudi (mean score 7.4) and non-Saudi parents (mean score 7.66; *P*=.54). Similarly, we found no significant relationship between knowledge scores and child performance in school (*P*=.59). All knowledge-related questions are presented in Table 2.

Mean parental attitude scores for each question are presented in Table 3. Parental attitudes were measured using the Likert scale, scored from 7 to 35, and the mean score was 26.51 (SD 4.284). The highest average positive attitude was obtained for the statement, “If there is more social support, my child will have more chances to self-develop” (4.3 out of 5). In contrast, the lowest average positive attitude was observed for the statement, “I do not feel that other people treat my child as different” (3.44 out of 5). There was no significant difference in attitude scores between men (mean 3.80) and women (mean 3.77; *P*=.77). Similarly, there was no difference in attitude according to the parent’s years of studying (*P*=.13) or according to the parent’s nationality (Saudi: mean 3.77 and non-Saudi: mean 3.82; *P*=.73). In addition, there was no significant association between the child’s age of diagnosis and parental attitude score (*P*=.49).

Mean parental behavioral scores for each question are presented in Table 4. The mean score for the behavioral section was 23.35 (SD 4.121). The statement with the highest mean score was “I allow my child to play sport” (4.17 out of 5), whereas the statement with the lowest mean score was “I can leave my child without supervision” (2.25 out of 5). There was no significant difference in behavioral score between men (mean 3.34) and women (mean 3.33; *P*=.99). Similarly, there was no difference in behavior according to the parent’s years of studying (*P*=.24) nor according to the parent’s nationality (*P*=.88) or according to the child’s performance in school (*P*=.96). The relationship between different sociodemographic characteristics and mean scores in knowledge, attitudes, and behavior are presented in the Tables 5 and 6.

**Table 1:** Demographic characteristics of parents and their children with epilepsy.

Characteristics			Values
**Parents**			
	**Gender, n (%)**		
		Male	65 (56.5)
		Female	50 (43.5)
	**Age (years), mean (SD)**		
		24-34	28 (24.3)
		35-44	55 (47.9)
		45-54	26 (22.6)
		≥55	6 (5.2)
	**Nationality, n (%)**		
		Saudi	77 (67.0)
		Non-Saudi	38 (33.0)
	**Education level, n (%)**		
		Primary school	7 (6.1)
		Intermediate school	16 (13.6)
		High school	33 (28.7)
		College	55 (47.8)
		None	4 (3.5)
**Children**			
	**Age (years), mean (SD)**		
		≤6	41 (35.7)
		7-14	50 (43.4)
		15-18	24 (20.9)
	**Education level, n (%)**		
		Primary school	26 (22.6)
		Intermediate school	9 (7.8)
		High school	15 (13.0)
		None	65 (56.5)
	**Performance, n (%)**		
		Excellent	11 (9.6)
		Needs improvement	26 (22.6)
		Good	13 (11.3)
		None	65 (56.5)
	**Age of diagnosis (years), n (%)**		
		<1	51 (44.3)
		1-4	26 (22.6)
		5-9	25 (21.7)
		10-14	6 (5.2)
		15-18	7 (6.1)
	**Who is taking care, n (%)**		
		Both parents	85 (73.9)
		Father	1 (0.9)
		Mother	28 (24.3)
		Sister	1 (0.9)

**Table 2:** Percentage of correct answers to questions comprising the knowledge score of parents of children with epilepsy.

Item	Correct answers (%)
Epilepsy is not an infectious disease	90.4
Epilepsy is not a psychiatric disease	73.0
Epilepsy is not, for the most part, hereditary	33.0
Not all the affected children lose consciousness during seizures	33.0
Not all the affected children have the same symptoms	71.3
Children with epilepsy are able to get vaccinated according to the current immunization calendar	63.5
Children with epilepsy, for the most part, do not have a lower IQ	47.0
Withdrawal of seizures after medication use does not mean that the patient is cured	76.5
If you skip therapy, next time, you should not take a double dose of medications	82.6
Children with epilepsy may encounter difficulties in learning	64.3
Onset of seizures may occur at any age	85.2
Diagnosis of epilepsy is usually made based on at least two unprovoked seizures	28.7

**Table 3:** Mean scores and standard deviations of attitude-related questions.

Question	Score, mean (SD)
I want my family and friends to know my child is epileptic	3.84 (1.196)
I do not feel that other people treat my child as different	3.44 (1.265)
I believe my child will be able to achieve success in his/her career	4.12 (0.984)
I am not worried about my child’s capability to make friends	3.75 (1.067)
I feel I receive enough support from my society regarding my epileptic child	3.49 (1.187)
I believe my child is as qualified as any other healthy children	3.57 (1.148)
If there is more social support, my child will have more chances to self-develop	4.30 (0.946)

**Table 4:** Mean scores and standard deviations of behavior-related questions.

Question	Score, mean (SD)
I am capable in taking care of my child during seizures	3.89 (1.145)
In general, I allow my child to participate in any activity he desires	3.64 (1.078)
I allow my child to play video games	3.06 (1.045)
I allow my child to play sport	4.17 (0.798)
Although my child is epileptic, I have time to meet other people and participate in other activities	4.06 (0.841)
I can leave my child without supervision	2.25 (1.091)
I allow my child to go on school trips	2.27 (1.126)

**Table 5:** Relationship between different sociodemographic characteristics and mean scores in knowledge, attitudes, and behavior, using independent samples *t* test and one-way analysis of variance.

Factor			Knowledge		Attitude		Behavior	
			Mean^a^	*P* value	Mean^b^	*P* value	Mean^b^	*P* value
**Parents**								
	**Gender**			.44		.77		.99
		Male	7.35		3.80		3.34	
		Female	7.66		3.77		3.33	
	**Nationality**			.54		.73		.88
		Saudi	7.40		3.77		3.34	
		Non-Saudi	7.66		3.82		3.32	
	**Education (years)**			.004		.13		.24
		<12	6.97		3.70		3.27	
		>12	8.09		3.88		3.40	
**Children**								
	**Education**			.51				.36
		Primary school	7.58		3.96	.23	3.49	
		Intermediate school	6.56		3.52	.23	3.33	
		High school	7.27		3.69	.23	3.40	
		Not studying	7.63		3.78	.23	3.26	
	**Age of diagnosis (years)**			.35		.49		.32
		<1	7.75		3.89		3.35	
		1-4	7.50		3.66		3.29	
		5-9	7.40		3.81		3.50	
		10-14	7.33		3.60		3.02	
		15-18	6.00		3.63		3.12	
	**Performance^c^**			.59		.18		.96
		Above average	7.13		3.90		3.44	
		Below average	7.46		3.71		3.43	
	**Primary caregiver**			.68		.76		.88
		Father	6.00		3.29		3.29	
		Mother	7.25		3.86		3.40	
		Both parents	7.56		3.77		3.31	
		Sister	9.00		3.71		3.57	

^a^Knowledge mean score out of 12.

^b^Attitudes and behavior mean score out of 5.

^c^Children who are not in school were excluded.

**Table 6:** Relationship between different sociodemographic characteristics and mean scores in knowledge, attitudes, and behavior, using Pearson correlation test.

Factor	Knowledge		Attitudes		Behavior	
	Pearson correlation	*P* value	Pearson correlation	*P* value	Pearson correlation	*P* value
Parental age	0.030	.75	−0.072	.44	0.004	.97
Child age	−0.068	.47	−0.188	.045	−0.016	.87

## Discussion

### Principal Findings

This study was conducted on parents to evaluate the knowledge, attitudes, and behaviors of parents toward their epileptic children; few parents answered all questions correctly (2/115, 2.0%). On the one hand, most of the participants (104/115, 90.4%) strongly think that epilepsy is not a communicable disease, and the mean score is less than 2 in previously conducted studies in Jordan (98.5%) and Serbia (99.5%) [20,21], yet slightly higher than that in a study conducted among Turkish parents (89.5%) and Iranian parents (83%) [22,23]. On the other hand, only 32% of parents in a study in Nigeria knew that epilepsy is not communicable [24]. This difference might be because of the variation in sociodemographic characteristics of other populations as well as a difference in educational quality. Most people still believe that the epilepsy is a mental disorder [25], but in our study, most parents knew it is not psychological (73%), compared with the Iranian study (60.2%) [23], Serbian study (68.1%) [21], and a study in Jordan (90.3%) [20].

Although most of our sample had a high level of education, there is still lack of knowledge and wrong beliefs about epilepsy, for example, 33% of parents think that epilepsy is mostly not hereditary, similar to the Serbian study in which 32% of parents answered correctly [21]. One possible cause of this misconception might be because of the high prevalence of consanguinity in our culture [26]. There is a significant relationship between the age of diagnosis and the knowledge about epilepsy; therefore, the earlier period of diagnosis might encourage the parent to read and know more about epilepsy to take care of their children properly and to make their life better.

In the attitudes section of the questionnaire, we found a positive response about the desire to inform friends and family members about their child who is suffering from epilepsy (mean score 3.84), suggesting that they already knew that the disease is not shameful. Serbian parents responded even more positively regarding that statement (mean score 4.3) [21]. Social support is essential to those children to help them defeat the frustrations and problems they might face and help enable them to be more productive in society. Moreover, our results showed that most of the parents believe that they have enough social support (mean score 3.49 out of 5); however, if they agreed that if they have more social support, their child will have better chances for self-development (mean score 4.30 out of 5). However, the Serbian parents agreed more strongly that their children already received enough social support (mean score 3.8 out of 5). They also agreed, to a lesser extent, that more social support will help their children grow and improve (mean score 3.2 out of 5) [21].

Behaviors of parents toward their children differ according to educational level, economic level, and cultural habits [27]. In our sample, we found that parents are supportive of activities that would help their children be happy and healthy, and this is a good result compared with Turkish mothers who were found to be less supportive [28]. We received a profoundly negative result about the possibility of leaving an epileptic child without supervision (mean 2.25), which could indicate greater parental responsibilities toward their children and an understanding about the disease and its sudden seizures so that it will give those children a good chance to avoid injuries during an unprovoked seizure.

One of the limitations of this study was the small sample size because of the high number of parents not answering the telephone as well as the poor cooperation and linguistic abilities of some parents. Furthermore, patients who did not answer the call or refused to participate may have a different set of knowledge, attitudes, and behaviors.

### Conclusions

In conclusion, the mean knowledge score of our sample was less than our expectation, and only 2 of 115 participants correctly answered all knowledge-related questions. As expected, we found a significant relationship between parental educational degree and knowledge score. Parental knowledge in our sample still needs improvement. Therefore, more awareness campaigns should be conducted for the community and for the parents of affected children to create a supportive environment for the children and help them thrive and develop and to help parents gain the skills to control their children's epilepsy to minimize the negative outcomes. Thankfully, multiple epilepsy-related educational programs around the world showed significant efficacy to correct misconceptions and improve parental knowledge toward their children’s condition [18,29-31], and the implementation of such programs should be considered in the future plans for improving knowledge in Saudi Arabia.

## Abbreviations

KAU: King Abdulaziz University
